# Pattern of Acute Intestinal Obstruction: Is There a Change in the Underlying Etiology?

**DOI:** 10.4103/1319-3767.70613

**Published:** 2010-10

**Authors:** Arshad M. Malik, Madiha Shah, Rafique Pathan, Krishan Sufi

**Affiliations:** Department of Surgery, Liaquat University of Medical & Health Sciences, Jamshoro, Pakistan

**Keywords:** Acute intestinal obstruction, adhesions, etiology, obstructed/strangulated hernias

## Abstract

**Background/Aim:**

To study the changing pattern of acute intestinal obstruction at a teaching institute.

**Patients and Methods:**

It is a prospective descriptive study conducted at a teaching hospital during the period from June 2004 to June 2009. All patients with clinical or radiological evidence of acute intestinal obstruction were included in this study regardless of the gender of the patient. Patients below the age of 10 years were excluded from the study. The treatment strategy was planned ranging from conservative treatment to emergency laparotomy after resuscitation and rehydration of the patient. Details of individual patients were recorded on a pro forma sheet and data analyzed statistically on SPSS version 14.

**Results:**

A total of 229 patients with acute intestinal obstruction were admitted and treated. The mean age of the study population was 43.08 ± 13.07 years. Postoperative adhesions accounted for 41% (*n*= 95) of the total cases, followed by abdominal tuberculosis (25%, *n*= 58), obstructed/ strangulated hernias of different types (18%, *n*= 42). There was an obvious change in the pattern of etiology of acute intestinal obstruction as the common causes were postoperative adhesions and abdominal tuberculosis instead of obstructed inguinal hernias.

**Conclusion:**

An increase in the adhesive obstruction and a concomitant decrease in the incidence of obstructed hernias indicate a changing trend towards early operation before it gets complicated. Abdominal tuberculosis is emerging as another common cause of acute bowel obstruction.

Acute intestinal obstruction is a common surgical emergency globally with high morbidity and mortality.[1–5] The most common underlying cause of acute bowel obstruction in the West has always been postoperative adhesions as suggested by many reports.[6–8] A number of studies conducted in our part of the world had found obstructed/ - strangulated hernias to be the most common underlying cause of acute intestinal obstruction.[9–10] This was attributed to a general reluctance for surgery due to unawareness, poverty and fear. During the last few years, a change in the etiology of acute intestinal obstruction has been noted in the developing countries.[11–16]

## PATIENTS AND METHODS

A prospective study of 229 patients, presenting with acute intestinal obstruction, admitted and treated at a teaching hospital over a period of five years from 2004 to 2009, was undertaken. All patients with radiological and clinical evidence of acute bowel obstruction admitted and treated in a surgical unit were included in this study regardless of the gender of the patients. However, patients below the age of 10 years and those with incarcerated and irreducible hernia and paralytic ileus were excluded from the study. All patients were admitted through casualty department and were received by two of the co-authors in the ward. Upon arrival of the patients in the ward, an immediate fluid and electrolyte resuscitation was started on every patient, and necessary investigations were done before surgery. Patients with previous laparotomy were initially put on conservative management comprising of nasogastric decompression, fluid and electrolyte correction by intravenous route and broad-spectrum antibiotics. Failure of relief of obstruction on this conservative treatment for more than 48 hours was followed by laparotomy. Patients with clinical suspicion and previous history of tuberculosis were also initially kept on conservative regime. The data collection was started by two of the co-authors immediately upon arrival of the patient in the ward.

The variables studied included demographic details, time between onset of symptoms and arrival in ward, symptoms and signs, imaging studies, initial resuscitation, type of treatment offered, operative findings, cause of obstruction and eventual outcome of the treatment offered. The data were collected on a pro forma sheet of individual patient and statistically analyzed using Statistical package for social sciences (*SPSS, version* 14.0; Chicago, IL, USA).

The ethical committee approval was not needed for this particular study.

## RESULTS

A total 229 patients with a mean age of 43.08 years (Std of 13.069) and a range of 13-74 presented with classical acute bowel obstruction during a period of five years and were included in this study. Males constituted 74% (*n*= 170) of the study population; and females, 26% (*n*= 59). Site of obstruction was found to be in the small bowel in a vast majority (85%) of the study population; while in 35 (15%) patients, the site of obstruction was in the large bowel. The most common features on presentation included distension of the abdomen (87%), vomiting (73%), absolute constipation (88%), dehydration (67%) and pain in abdomen (75%). In majority of the patients, there was a substantial delay in admission to the hospital from the time of development of the symptoms, as shown in Table 1. Preliminary investigations included complete blood picture, plain x-ray abdomen (erect and supine films), ultrasound of abdomen, serum electrolytes and urea. There were multiple air-fluid levels on plain x-ray films in 87% of the patients. We found concomitant pulmonary tuberculosis in 2 patients who presented with acute intestinal obstruction due to abdominal tuberculosis. Patients with adhesive obstruction and suspected abdominal tuberculosis were given a trial of conservative treatment for 48 to 72 hours. Of these, 76% responded with complete recovery, but the remaining 34% needed exploration due to worsening condition. Overall 192 patients were operated, and the various underlying causes of obstruction discovered in relation to the site of obstruction are shown in Table 2. There was a surprisingly high proportion of adhesive obstruction followed by tuberculosis of abdomen. This is contrary to the results obtained a couple of decades back in the same setting, when obstructed hernias were the most common cause of acute bowel obstruction. Depending upon the underlying cause of the obstruction, various treatment modalities were adopted, ranging from conservative measures to resuscitation followed by laparotomy and resection anastomosis wherever indicated. Of the total number of patients, 200 recovered completely while 3 patients developed fecal fistula, 15 patients developed wound infection and 3 patients developed wound dehiscence. There was an overall mortality of 3.49%. Mortality was high in patients who were brought too late to our hospital (*P* < 0.001) after the onset of the symptoms, as shown in Table 3.

**Table 1:** Duration of symptoms

Duration of symptoms	No. of patients	Percentage
1 day	08	3.49
2-5 days	64	27.94
One week	153	66.81
Two weeks and above	04	1.74
Total	229	99.99

**Table 2:** Underlying cause and site of obstruction

Underlying cause	Site of obstruction		
	Small bowel	Large bowel	Total
Obstructed/strangulated inguinal hernia	43	01	44
Postoperative adhesions	94	00	94
Abdominal tuberculosis	55	00	55
Intra-peritoneal bands	11	02	13
Obstructed paraumbilical hernia	05	00	05
Volvulus	03	06	09
Left-sided colon cancer	00	05	05
Obstructed paraumbilical hernia	01	00	01
Obstructed/strangulated femoral hernia	03	00	03
Total	215	14	229

**Table 3:** Outcome of treatment in relation to duration of symptoms

Duration of symptoms	Outcome of treatment					Total
	Complete recovery	Fecal fistula and re-operation	Wound infection	Wound dehiscence	Died
1 day	07	00	00	01	00	08
2-5 days	51	02	08	01	00	62
One week	142	01	07	01	03	154
Two weeks and above	00	00	00	00	05	05
Total	200	03	15	03	08	229

P < 0.001						

## DISCUSSION

Acute intestinal obstruction is one of the common lifethreatening emergencies all over the world.[1,17–19] There is a global change in the spectrum of etiology of acute intestinal obstruction over the past few years. A number of recent studies have found adhesive obstruction to be replacing obstructive hernias as the most common cause.[20–22] This study is done to explore a similar change in the spectrum of acute intestinal obstruction in our part of the developing world, comparing the pattern of etiology at present with results obtained in the past. The pattern of presentation in our study is consistent with that in the reports of many similar studies, and the same is true for the sex distribution.[23–26]

The mean age of 43.08 years in our study is also consistent with age incidence in many similar reports.[12,26–27] The most common underlying cause of obstruction in this study is found to be adhesive obstruction. This is contrary to the earlier results in our region, when obstructed inguinal hernia was the commonest cause of acute intestinal obstruction.[9,26] It was believed that patients were reluctant for elective surgery of hernias due to poverty, lack of education, and general fear of surgery. This led to a large number of hernias presenting as obstructed/ strangulated bowel obstruction. A growing knowledge about hernias and rising fear of likely complications are probably the reasons for an increasing number of hernias repaired electively. There is also a parallel increase in the number of laparotomies, and this has raised the incidence of adhesive obstruction in our institute. Maximum numbers of patients with adhesive obstruction (60%, *n*= 57) in our study had a history of appendectomy and other abdominal operations during the last six months to one year. Similar results are also described by many authors who have conducted similar trials.[12,27] All patients with adhesive obstruction were initially given a trial of conservative treatment, and this approach is recommended and adopted by many other authors in their trials.[28,30] A total of 76% of patients of adhesive obstruction responded with complete recovery in our study.

Another important and increasingly common cause of acute bowel obstruction is found to be abdominal tuberculosis. Our results show a total of 55 (24%) patients of abdominal tuberculosis presenting as acute intestinal obstruction. This observation is comparable to that in many similar local studies in our country and in neighboring countries.[31] There is an overall mortality of 3.49%, which is found directly related to the delay between onset of obstructive symptoms and arrival at our hospital (*P* < 0.001). This delayed presentation increases morbidity and mortality many-folds, as is evident from our results.

In conclusion, we have found that adhesions are becoming an ever increasing underlying cause of bowel obstruction. A trend of elective hernia surgery has reduced the number of patients of hernias presenting with obstruction of bowel. Acute bowel obstruction due to abdominal tuberculosis is becoming a common occurrence in our region.
